# Children’s level of word knowledge predicts their exclusion of familiar objects as referents of novel words

**DOI:** 10.3389/fpsyg.2015.01200

**Published:** 2015-08-11

**Authors:** Susanne Grassmann, Cornelia Schulze, Michael Tomasello

**Affiliations:** ^1^Department of Developmental Psychology, University of ZurichZurich, Switzerland; ^2^Department of Developmental and Comparative Psychology, Max Planck Institute for Evolutionary AnthropologyLeipzig, Germany; ^3^Christoph-Martin-Wieland-Postdoc-Fellow, University of ErfurtErfurt, Germany

**Keywords:** mutual exclusivity, principle of contrast, exclusion inference, word learning, word knowledge, production, comprehension, label retrieval

## Abstract

When children are learning a novel object label, they tend to exclude as possible referents familiar objects for which they already have a name. In the current study, we wanted to know if children would behave in this same way regardless of how well they knew the name of potential referent objects, specifically, whether they could only comprehend it or they could both comprehend and produce it. Sixty-six monolingual German-speaking 2-, 3-, and 4-year-old children participated in two experimental sessions. In one session the familiar objects were chosen such that their labels were in the children’s productive vocabularies, and in the other session the familiar objects were chosen such that their labels were only in the children’s receptive vocabularies. Results indicated that children at all three ages were more likely to exclude a familiar object as the potential referent of the novel word if they could comprehend and produce its name rather than comprehend its name only. Indeed, level of word knowledge as operationalized in this way was a better predictor than was age. These results are discussed in the context of current theories of word learning by exclusion.

## Introduction

Even very young children felicitously infer the referents of novel words. For example, when asked to fetch “the modi” (with “modi” being a new word) in the presence of objects for which they already know names and one object that they do not know yet, infants exclude the familiar objects as potential referents of the new word and take the speaker to refer to the previously unknown object. Such avoidance of lexical overlap by exclusion inference acts as a powerful constraint to zero in on a speakers’ intended referents in a variety of word learning situations (cf. Markman, 1989; Golinkoff et al., 1992).

Vincent-Smith et al. (1974) were the first to demonstrate this phenomenon in a controlled experiment in 20- to 31-month-old children. Subsequently, the phenomenon, also dubbed as the disambiguation effect, has been demonstrated not only for common names (e.g., Markman and Wachtel, 1988; Gathercole, 1989; Markman, 1989; Merriman and Bowman, 1989; Clark, 1990; Golinkoff et al., 1992; Diesendruck and Markson, 2001), but also for other referential terms, ranging from proper names (Diesendruck, 2005) to adjectives (Carey and Bartlett, 1978) to verbs (Merriman et al., 1996), and referential facts (Diesendruck and Markson, 2001; Kalashnikova et al., 2014).

Avoidance of lexical overlap has been demonstrated in infants as young as 10–19 months of age (Graham et al., 1998; Halberda, 2003; Markman et al., 2003; Mather and Plunkett, 2010; Song et al., 2014). However, older children and children with larger vocabularies avoid lexical overlap more reliably. For example, Merriman and Bowman (1989) found that 4-year-old children excluded familiar objects as referents of novel words more reliably than 2-year-old children (see also Merriman and Schuster, 1991). Other studies demonstrated that within one age group, children with larger vocabularies (both receptive and expressive vocabulary) avoided lexical overlap more reliably than did their age peers with smaller vocabulary size (Mervis and Bertrand, 1994; Graham et al., 1998; Law and Edwards, 2014).

One interpretation that intuitively comes to mind to explain the effects of age and vocabulary size would be that older children as well as children with larger vocabularies know objects and labels better and this is what leads to better exclusion. Thus, if children see an object for which they know the familiar label less well, this label might be difficult to retrieve, or it may only be weakly activated, which then results in less reliable exclusion of the corresponding object as a potential referent of a novel word (cf., Merriman and Marazita, 1995 for a similar argument). Indeed, studies by Merriman and Marazita (1995) and Merriman (1999) suggest that the avoidance of lexical overlap depends on the processes of object identification, label retrieval, comparison of the novel word with the retrieved lexical entries, and mismatch detection (cf., Grassmann, 2013). The more difficult these processes, the less likely children are to avoid lexical overlap. For example, the phonological form of the novel word and the typicality of the familiar object influence the likelihood of lexical overlap: Children are less likely to avoid lexical overlap if the familiar object is an atypical exemplar of a known category or if the novel word is highly similar to words in the children’s vocabulary (Merriman and Schuster, 1991; Merriman and Marazita, 1995).

Therefore, in the current study, we asked whether children’s level of word knowledge of a particular familiar object is relevant to their likelihood of excluding this very object as the referent of a novel word independently of their age. The rationale for this was that retrieval of less well known object labels should be more difficult and thus exclusion of a corresponding familiar object as the referent of a novel word less likely. However, word knowledge increases gradually (Fernald et al., 2006; McMurray et al., 2012; Smith et al., 2014) and thus label retrieval should develop gradually as well. As an approximation, the children’s individual level of word knowledge was operationalized in terms of whether the children were able to spontaneously produce a label for a certain object (high level of word knowledge), produce it upon request (medium level of word knowledge), or only comprehend it (low level of word knowledge). If the retrieval hypothesis is correct, even young children should show very reliable exclusion of familiar objects for which they can spontaneously retrieve and produce a label. In addition, the more difficult the retrieval of a familiar label is for children, the less reliably the children should exclude the corresponding familiar objects as referents of novel words. Indeed, even 4-year-olds might not exclude familiar objects with less well known labels reliably.

## Materials and Methods

### Participants

Sixty-nine monolingual German-speaking 2-, 3-, and 4-year-old children participated in the study. Three children had to be excluded from analysis (two 2-year-olds because they had not understood the game or were uncooperative and one 4-year-old because she turned out to be bilingual). Thus, the final sample comprised 66 children (2-year-olds: *n* = 23; 11 girls, 12 boys; mean age = 2;1,29; range = 2;0,4 to 2;3,29 / 3-year-olds: *n* = 22; 11 girls, 11 boys; mean age = 3;2,4; range = 3;0,3 to 3;3,28 / 4-year-olds: *n* = 21; 12 girls, 9 boys; mean age = 4;6,0; range = 4;4,5 to 4;8,0). This study was carried out in accordance with the ethical standards for research with children and in accordance with the laws and rules governing psychological research in Germany.^1^ The children’s parents had previously volunteered to participate in studies of child development. The parents of all children gave written informed consent. The children received a small gift for participation.

### Materials and Design

All children participated in two experimental sessions. Each session comprised six object choice trials. In one session the familiar objects were chosen such that their labels were likely to be in the children’s productive vocabularies (Highly Familiar Objects Session). Thus, children of all age groups saw the same objects in the Highly Familiar Objects Session: a car, a shoe, a chair, a banana, an apple, and a tree. In the other session, the familiar objects were chosen such that their labels were likely to be only in the children’s receptive vocabularies (Less Familiar Objects Session). Thus, the less familiar objects differed across the three age groups. This was done in order to ensure that there are objects among the familiar objects for which children of different ages are less likely to spontaneously retrieve a familiar label (2-year-olds: a brush, a hammer, a ladle, a mushroom, a saw, and a sponge; 3-year-olds: tongs, file, wrench, coat hanger, spin top, and a lock; 4-year-olds: strainer, thermometer, microphone, can opener, corkscrew, and tweezers.). All familiar objects were determined in the pre-test (see Supplementary Tables SA–SD).

Each familiar object was paired with a novel object. As novel objects we used an unusual yoyo, a U-shaped door stopper, a cone-shaped plastic piece, five different curtain rod finials, a modified bird toy, an eﬄux filter with plush, the glittery part of a kaleidoscope, and a bike reflector. These objects were selected from a pretested pool of novel objects that are unlikely to be labeled by 2- to 4-year-old children. Twelve phonotactically correct nonce-words (‘*nohle*,’ ‘*tahne*,’ ‘*doffe*,’ ‘*siehle*,’ ‘*kulde*,’ ‘*fende*,’ ‘*albe*,’ ‘*mehfe*,’ ‘*losse*,’ ‘*puhne*,’ and ‘*welne*’) were randomly assigned to the pairs of one novel and one familiar object. The pairing of the familiar and the novel objects and the order of presentation of the pairs, and the left–right position of the novel object was counterbalanced.

The two sessions were run on two successive days (for 12 children the second session was run 2 days after the first session). The order of condition was counterbalanced across children.

In each session a picture book was used to test the children’s individual productive or receptive knowledge of the familiar objects’ labels. The 2-year-olds’ picture books consisted of four photographs of real objects per page. That is, each page depicted two of the (highly or less) familiar objects used in the session and two additional familiar objects that were not used in the study. The 3- and 4-year-olds’ picture books differed from the picture books used with the 2-year-olds in that a photograph of one additional novel object – that was also not used in the study – was included per page.

### Procedure

The children were tested individually in a quiet room in their daycare centers. During the study the child sat at a table with the Experimenter (E) directly across. Prior to each of the two sessions, E and the child played together until the child felt comfortable with E. Each session started with two warm-up trials to familiarize children with the structure and rationale of the object selection game. Then the six experimental trials were conducted. At the end of both sessions the children’s individual level of knowledge of the familiar objects’ labels was assessed. All sessions were videotaped and later coded from tape.

#### Warm-up

Experimenter held two familiar animals (e.g., elephant and giraffe) approximately shoulder width apart in children’s eye height and said, “Look what I have here.” Then E put the two objects on the table. While she was still holding both objects, she said, “Let’s play with the elephant!” After that E held her open hand in the middle between the two objects and said, “Give me the elephant.” E looked straight at the child throughout the presentation and the request of the object. If a child did not respond immediately, E repeated her request. And when a child picked the wrong animal, E said, “No, this is not the elephant” and repeated her request. The rationale of this corrective feedback was to ensure that the children responded to E’s request and not to individual preferences. After the child picked the requested object, E said, “Thank you. I’ll show you what this can do.” E then demonstrated an action and the child and E played for approximately 30 s with the animal. The rationale for this was to introduce the object choice task in a game-like and pragmatically normal interaction. The procedure was repeated for a second set of toy animals^2^.

#### Object Selection

The procedure was identical to the Warm-up trials, except that E presented a pair of one novel and one familiar object (position counterbalanced) to the child and no feedback was given about the child’s choice. Rather, when the child had selected an object, E responded in a neutral manner independently of whether the child had chosen the novel or the familiar object and said, “Thank you, I’ll show you how to play with this.” E then demonstrated an action and the child and E played for approximately 30 s with the selected toy (e.g., spinning it or sliding it back and forth, etc.). The procedure was repeated for a total of six trials per session.

#### Label Knowledge Test

After the object choice task, the children’s level of word knowledge of the familiar objects’ labels was administered. To this end, the child and E looked at a picture book together. The picture book depicted photographs of the familiar objects used in the session and additional distractor pictures (see Materials and Methods section). At first, the children were asked to label the objects on each page, by saying, “Look there. What do you see on this page? Do you know any of these items?” For the session’s familiar objects that the child had not yet named spontaneously, E subsequently asked the child, “Can you tell what this is?” and individually pointed to the objects that the child had not yet labeled. This was done for each page of the picture book. Thus, children’s labeling of objects upon request differed from their spontaneous labeling events in that they had more time to come up with the appropriate word and that E’s pointing gesture helped them focus their attention to the object under discussion (rather than processing the visual information of all pictures on the page). Having completed a first inspection of the picture book, E told that child that they would look through the book again. Then, the children’s receptive knowledge of object labels was tested by asking for example, “Can you show me the shoe?”

### Coding and Reliability

#### Object Choice

The children’s object choice was coded as “novel” or “familiar.” Objects counted as chosen when the children picked one object up and handed it to E, when they held up one object to E, or when they pushed one object on the table toward E.

#### Label Knowledge

The children’s individual level of word knowledge was coded as “spontaneously labeled” (when the label for the familiar object was produced during the object choice test or in the first phase of the picture book reading), “labeled upon request” (when the label for the familiar object was produced upon the experimenter’s direct question and pointing to one object), “comprehension only” (when the children did not produce any label but identified the familiar object correctly when asked to point to it) or “unknown.” The latter occurred when a child did not know a label for the familiar object and these trials were excluded from analysis (76 trials across all children, see Supplementary Table SE).

#### Reliability

Three additional coders scored four children each (one coder per age group). As estimated by Cohen’s Kappa, inter-observer reliability for object choice was 1 for all age groups. For label knowledge the inter-observer reliability was κ = 0.787 for 2-year-olds, κ = 0.898 for 3-year-olds and κ = 0.931 for 4-year-olds.

## Results

For statistical analysis the dependent variable was how often the children chose the novel object upon hearing a novel word. Since we were interested in the question whether knowledge of the familiar object’s label influences children’s object choice, 76 trials were excluded from analysis, because the familiar object was unknown to the child. Another 18 trials were excluded from analyses, because the trial’s novel object was labeled. The reason for excluding those trials was that the study’s intended object knowledge status (novel object without a label vs. familiar object with a label) is unclear in cases were children either labeled a novel object or did not show any word knowledge concerning the familiar object. Preliminary analysis revealed that gender and trial number had no effect on children’s novel object selection and was thus not regarded further.

Similar to previous research we first analyzed the children’s novel object selection averaged across all trials independently of the children’s level of word knowledge. Results show that while all age groups chose novel objects significantly above chance [2-year-olds: *M* = 68.2%, SD = 18.8%, *t*(22) = 4.653, *p* < 0.001, Cohen’s *d* = 1.984; 3-year-olds: *M* = 71.4%, SD = 23.5%, *t*(21) = 4.267, *p* < 0.001, Cohen’s *d* = 1.862; 4-year-olds: *M* = 75.9%, SD = 27.2%, *t*(20) = 4.367, *p* < 0.001, Cohen’s *d* = 1.953], no age effect was found [*F*(2,63) = 0.601, *p* = 0.551, η^2^ = 0.019].

**FIGURE 1:**
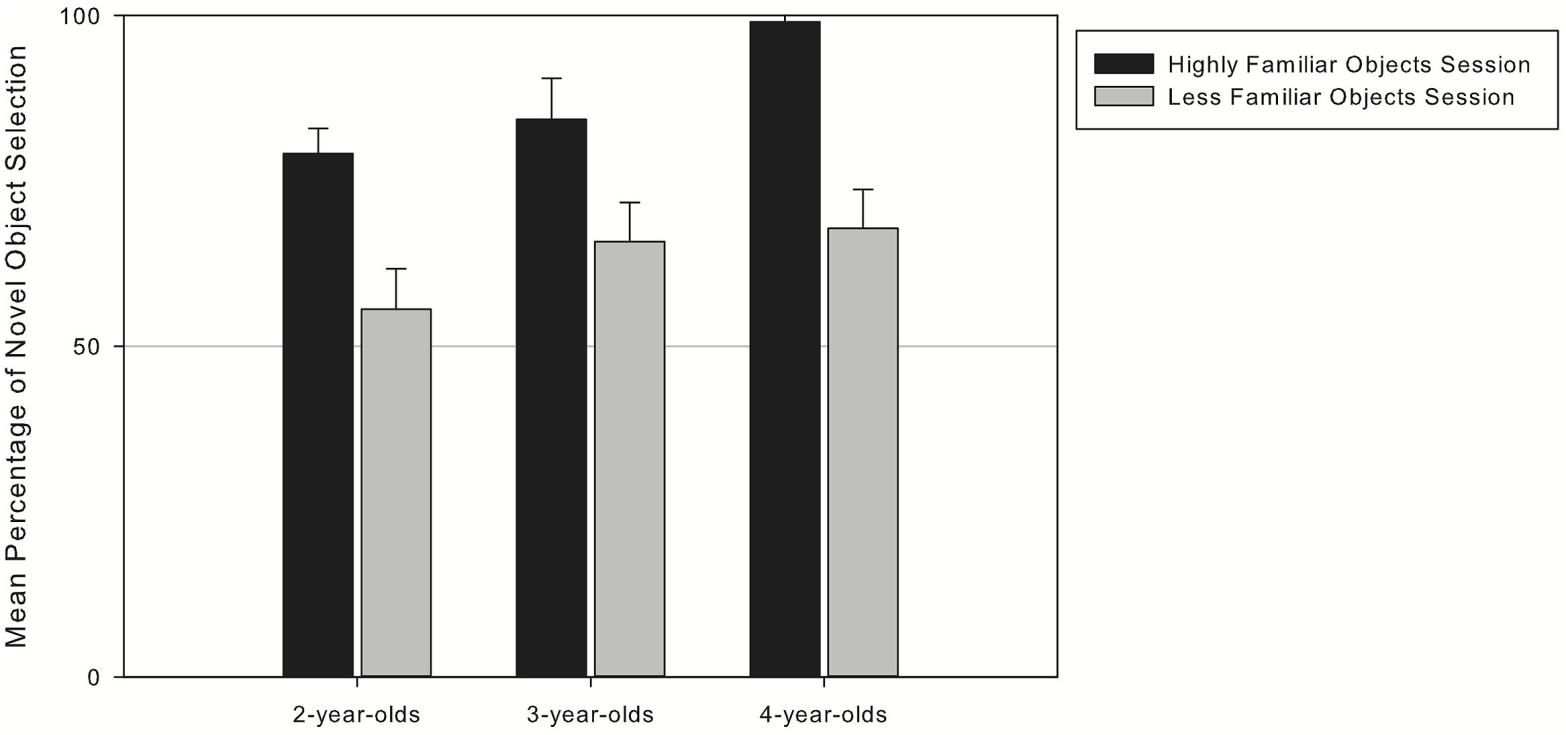
**Children’s novel object selection in the two sessions per age**.

Since the children participated in two sessions, in the next analysis we compared the children’s novel object selection across the two sessions (**Figure 1**). A repeated measurements *t*-test revealed that the children collapsed across age groups were more likely to choose the novel object in the Highly Familiar Objects Session (*M* = 87.1%, SD = 18.9) than in the Less Familiar Objects Session [*M* = 62.6%; SD = 25.5, *t*(52) = 6.760, *p* < 0.001, Cohen’s *d* = 1.065]. An additional 2 (sessions) × 3 (age) ANOVA that checked for this effect in all age groups separately found no interaction of age and session [*F*(2,50) = 0.97, *p* = 0.386, η^2^ = 0.02]. However, we found a significant but small effect for age (children show better exclusion when they get older [*F*(2,50) = 4.001, *p* = 0.024, η^2^ = 0.14] and a significant effect of medium effect size for session [*F*(2,50) = 45.017, *p* < 0.001, η^2^ = 0.46]. Subsequent *t*-tests separately for each age group reveal that the children of all age groups show better exclusion in the Highly Familiar Objects Session compared to Less Familiar Objects Session [2-year-olds: *t*(19) = 3.690, *p* = 0.002, Cohen’s *d* = 1.008; 3-year-olds: *t*(15) = 2.875, *p* = 0.012, Cohen’s *d* = 0.77; 4-year-olds: *t*(16) = 5.316, *p* < 0.001, Cohen’s *d* = 1.746]. Nevertheless the children chose novel objects above chance level (50%) in both sessions^3^ [HF: *M* = 85.3%, SD = 20, *t*(57) = 13.455, *p* < 0.001, Cohen’s *d* = 3.564; LF: *M* = 60.3%, SD = 27.7, *t*(60) = 2.907, *p* = 0.005, Cohen’s *d* = 0.751].

### Analyses based on the Children’s Individual Level of Word Knowledge

We used a binary logistic regression to investigate our main question of whether and how strongly the children’s age (three levels) and individual word knowledge of the familiar object (three levels) were predictive of novel object selection. The three age levels were: 2-year-olds, 3-year-olds, and 4-year-olds. The three levels of word knowledge level were ‘comprehension only’ (level 1, 145 trials), ‘labeled upon request’ (level 2, 85 trials) and ‘spontaneously labeled’ (level 3, 390 trials). **Table 1** summarizes the children’s object choice. For both predictor variables contrast estimates were set such that step-wise comparisons to the lowest level of each predictor variable (comprehension only and 2-years of age) was possible.

**Table 1:** Children’s novel object selection in the trials of each level of word knowledge per age group.

	2-year-olds		3-year-olds		4-year-olds	
	Novel object selection (total)^∗^	%	Novel object selection (total)^∗^	%	Novel object selection (total)^∗^	%
Comprehension only	33 (65)	50.77	17 (34)	50.00	19 (46)	41.30
Labeled upon request	31 (44)	70.45	13 (22)	59.09	17 (19)	89.47
Spontaneously labeled	85 (106)	80.19	122 (154)	79.22	122 (130)	93.84

*^∗^Raw number of trials in which the children chose the novel object. The number in brackets is the total number of trials per age group and level of word knowledge.*

The first logistic regression predicted infants’ novel object selection using age, level of word knowledge and an interaction between those two predictor variables as factors. Results indicated that the whole model was significant [χ^2^(8) = 91.578, *p* < 0.001]. Moreover, the whole model explained 20.1% of our data (*Nagelkerke R*^2^ = 0.201). The contributions of each factor and the interaction can be seen in **Table 2**.

**Table 2:** Predictor variables (age, level of word knowledge) and interaction in binary logistic regression.

	Variables in the Equation						95% confidence interval	
	*B*	SE	Wald	*df*	*p*	Odds ratio	Lower	Upper
Age			7.147	2	0.028			
Age(1)	-0.197	0.253	0.609	1	0.435	0.821	0.500	1.347
Age(2)	0.738	0.335	4.850	1	0.028	2.093	1.085	4.037
Word knowledge			67.095	2	0.000			
Word knowledge(1)	1.232	0.354	12.153	1	0.000	3.430	1.715	6.858
Word knowledge(2)	1.927	0.236	66.735	1	0.000	6.870	4.327	10.909
Age ^∗^ word knowledge			12.134	4	0.016			
Age(1) by word knowledge(1)	-0.471	0.690	0.465	1	0.495	0.625	0.161	2.416
Age(1) by word knowledge(2)	-0.029	0.527	0.003	1	0.956	0.971	0.346	2.730
Age(2) by word knowledge(1)	1.653	0.905	3.336	1	0.068	5.224	0.886	30.790
Age(2) by word knowledge(2)	1.709	0.586	8.491	1	0.004	5.521	1.750	17.424
Constant	0.946	0.129	53.461	1	0.000	2.576		

*Age(1) = one step up in age (from 2-year-olds to 3-year-olds); age(2) = two steps up in age (from 2-year-olds to 4-year-olds); word knowledge(1) = one step up in level of word knowledge (from “comprehension only” to “labeled upon request”); word knowledge(2) = two steps up in level of word knowledge (from “comprehension only” to “spontaneously labeled”).*

In order to estimate the independent effects of age, level of word knowledge and their interaction we ran further logistic regressions. First, the interaction of age and word knowledge explains only 3.3% of our data [*Nagelkerke R*^2^ = 0.033; χ^2^(4) = 13.952, *p* = 0.007]. This interaction suggests that the effect of word knowledge is different at different ages (see **Table 3**).

**Table 3:** Interaction of age and level of word knowledge as independent predictor variable.

	Variables in the Equation						95% confidence interval	
	*B*	SE	Wald	*df*	*p*	Odds ratio	Lower	Upper
Age ^∗^ word knowledge			13.355	4	0.010			
Age(1) by word knowledge(1)	-1.515	0.767	3.904	1	0.048	0.220	0.049	0.988
Age(1) by word knowledge(2)	-0.743	0.542	1.880	1	0.170	0.476	0.164	1.376
Age(2) by word knowledge(1)	0.568	0.702	0.655	1	0.418	1.765	0.446	6.990
Age(2) by word knowledge(2)	1.194	0.509	5.514	1	0.019	3.302	1.218	8.947
Constant	1.084	0.096	127.605	1	0.000	2.956		

*Age(1) = one step up in age (from 2-year-olds to 3-year-olds); age(2) = two steps up in age (from 2-year-olds to 4-year-olds); word knowledge(1) = one step up in level of word knowledge (from “comprehension only” to “labeled upon request”); word knowledge(2) = two steps up in level of word knowledge (from “comprehension only” to “spontaneously labeled”).*

The regression with age as single predictor variable explains about 2% of or data [χ^2^(2) = 8.012, *p* = 0.018; *Nagelkerke R*^2^ = 0.019]. A detailed inspection of the regression coefficients suggests that the age effect is driven by the 4-year-old children who, compared to 2-year-olds, were 1.9 times more likely to select novel objects [*Wald*(1) = 7.357, *p* = 0.007, *Exp(B)* = 1.892; see **Table 4**].

**Table 4:** Age as independent predictor variable.

	Variables in the Equation						95% confidence interval	
	*B*	SE	Wald	*df*	*p*	Odds ratio	Lower	Upper
Age			7.645	2	0.022			
Age(1)	0.149	0.214	0.487	1	0.485	1.161	0.764	1.765
Age(2)	0.637	0.235	7.357	1	0.007	1.892	1.193	2.998
Constant	1.076	0.094	131.948	1	0.000	2.934		

*Age(1) = one step up in age (from 2-year-olds to 3-year-olds); age(2) = two steps up in age (from 2-year-olds to 4-year-olds).*

In a final regression we found that the children’s individual level of word knowledge significantly predicts children’s object choice [χ^2^(2) = 70.058, *p* < 0.001], and 15.7% of our data can be explained by the participants’ level of word knowledge alone (*Nagelkerke R*^2^ = 0.157). Interestingly, a closer inspection of the contrast within the variable reveals that being able to label the familiar object (compared to only comprehending the familiar object’s label) yields 2.8 times more novel object selection. Even more strikingly, being able to spontaneously label the familiar object yields nearly six times more novel object selection compared to only comprehending the alternative’s familiar object label (see **Table 5**).

**Table 5:** Level of word knowledge as independent predictor variable.

	Variables in the Equation						95% confidence interval	
	*B*	SE	Wald	*df*	*p*	Odds ratio	Lower	Upper
Word knowledge			67.434	2	0.000			
Word knowledge(1)	1.029	0.293	12.364	1	0.000	2.800	1.577	4.969
Word knowledge(2)	1.782	0.217	67.430	1	0.000	5.941	3.883	9.089
Constant	0.840	0.108	60.463	1	0.000	2.317		

*Word knowledge(1) = one step up in level of word knowledge (from “comprehension only” to “labeled upon request”); word knowledge(2) = two steps up in level of word knowledge (from “comprehension only” to “spontaneously labeled”).*

## Discussion

It is well established that when inferring the referents of novel words, children exclude objects for which they already know a label. In the current study we found that 2-, 3-, and 4-year-olds were more likely to avoid lexical overlap and thus chose the novel object when they actively produced the label of the familiar object (compared to only comprehending the familiar object label). Indeed, regression analyses revealed that the children’s level of familiar word knowledge explained 16% of the variance in the data and was a better predictor of their object choice than age or an interaction between age and word knowledge.

This finding adds to previous research which demonstrated effects of age and vocabulary size on children’s exclusion of familiar objects as referents of novel words (Merriman and Bowman, 1989; Merriman and Schuster, 1991; Mervis and Bertrand, 1994; Graham et al., 1998; Law and Edwards, 2014). In fact, the current findings might even provide an explanation for these effects: It is possible that the older children and the children with larger vocabularies in these earlier studies simply knew the task-relevant words better. This is likely because in all of the previous studies, the same material was used for children of different ages or vocabulary sizes and the researchers only ensured the children’s individual knowledge of the task-relevant words was receptive word knowledge. Therefore, although all children in previous studies knew the relevant familiar object labels at least receptively, older children and children with larger vocabularies likely knew the words better.

The current findings are particularly interesting regarding process models of the phenomenon (cf., Merriman and Marazita, 1995; Merriman, 1999; Grassmann, 2013). These models suggest that in order to determine the referent of a novel word in a disambiguation task, children first retrieve the familiar object’s label from long-term to working memory, then compare it with the working-memory phonological representation of the novel word, and finally need to detect a mismatch to exclude familiar objects. The current study is indecisive as to whether the level of word knowledge influences the retrieval of a label or the comparison of the retrieved label of the familiar object’s label with the spoken novel word – or both. According to the label retrieval hypothesis less well known words (in the current study: words that children could only comprehend but not actively produce) might be difficult to retrieve upon seeing a corresponding object. In this case, the children’s weaker exclusion of familiar objects with less well known labels as referents of novel words could be explained as a failure to retrieve the familiar object’s label – and thus no comparison process is initiated. However, cross-model priming studies suggest that even less well known words are retrieved from long-term memory and produce a phonological incongruency effect in adults and infants as young as 14 months of age (Friedrich and Friederici, 2004, 2005). Interestingly, however, adults and 19-month-olds with stronger knowledge of the task-relevant words subsequently show a semantic incongruency effect as well. That is, when a spoken word (novel or familiar) is incongruent to the label of a depicted familiar object, adults and 19-month-olds with better word knowledge show a N400 response. This N400 is absent in 14-month-olds and in 19-month-olds with less word knowledge (Friedrich and Friederici, 2004, 2006). It seems likely that these processes of phonological and semantic mismatch detection play a role in children’s avoidance of lexical overlap in reference resolution. Future research is necessary to determine the role that label retrieval and phonological and semantic mismatch detection play in children’s avoidance of lexical overlap.

Further, our finding that level of word knowledge influences children’s avoidance of lexical overlap seems to conflict with earlier demonstrations of the exclusion of objects with newly learned labels. That is, previous research demonstrated that children exclude objects for which they just learned a label as referents of novel labels (e.g., Markman and Wachtel, 1988; Diesendruck and Markson, 2001; Diesendruck, 2005; Song et al., 2014). This effect seems difficult to reconcile with the current findings, since word knowledge is build up incrementally (Fernald et al., 2006; McMurray et al., 2012; Smith et al., 2014) and it is thus highly unlikely that a newly learned word is as well known and as easily retrieved as a well known word in a child’s productive vocabulary.

We suggest that the seeming contradiction between the current findings and studies demonstrating avoidance of lexical overlap with newly learned labels can be resolved by distinguishing situation time and developmental time (cf., Kucker et al., 2015): In studies such as Diesendruck and Markson’s (2001), the relevant time dimension is the situational time. That is, the children in these studies learned a novel label for a novel object and then were presented with an object pair comprising the formerly novel object and a “novel–novel” object and they heard a “novel–novel” word. The phonological representations necessary for blocking the “familiar” (formerly novel) object as the referent of the “novel–novel” label are built and activated in working memory in the situation time. It is thus not surprising that the children chose the “novel–novel” object even though they had just learned the novel label. In contrast, in the current study, the children’s word knowledge in developmental time was relevant, and thus the retrieval of object labels into working memory depended solely upon the children’s (attempt to) access labels from long-term memory.

The current findings are compatible with all theoretical accounts as to why children exclude familiar objects as referents of novel labels. Although these accounts do not explicitly address the question of label retrieval (besides Merriman and Marazita, 1995; Grassmann, 2013), we suggest to integrate our current findings into the existing accounts in the following ways: First, according to lexical gap accounts (e.g., Golinkoff et al., 1992), children need to identify objects for which they do not know a label. In order to do so it is necessary to attempt to retrieve labels for all available candidate referents. With respect to this account, the current findings suggest that when retrieval is difficult – as in the case of less well known words – and fails, familiar objects might falsely be identified as nameless and lead to errors in referent identification. Second, according to pragmatic accounts (Clark, 1990; Diesendruck, 2005), children reason about speaker’s referential choices based on the expectations of conventional word use. We suggest that in order to expect a speaker to use a conventional label for a familiar object, children first need to retrieve the familiar objects’ label themselves (cf., Grassmann, 2013). Thus, when children are able to produce a certain familiar word, they are better able to expect another speaker’s conventional word use. Third, although the current findings are also compatible with the Mutual Exclusivity account (Markman, 1989; Merriman and Bowman, 1989), it is less clear that this account would expect an effect of word knowledge/label retrieval. According to the Mutual Exclusivity account, children exclude familiar objects as referents of novel words because they assume that each object belongs to one category and that each category has one label. Thus, strong knowledge of a category label might not be necessary from their theoretical perspective (the label-slot of the category is filled whether the label is known receptively only or in the child’s productive vocabulary). Nevertheless, Merriman and Marazita (1995) suggest that label retrieval and label comparison are two very important processes in the application of Mutual Exclusivity. Since the current findings are compatible with all theoretical accounts to children’s avoidance of lexical overlap, we suggest that it is time to move beyond such either-or accounts and focus more on the exact processes underlying the avoidance of lexical overlap.

## Conclusion

The current study demonstrated that 2-, 3-, and 4-year-old children are more likely to exclude familiar objects as referents of novel labels when they can actively produce the label of the familiar object. This finding has important implications for future research and suggests that the material has to be very carefully chosen and the children’s individual level of word knowledge has to be established. The finding also suggests interesting new directions for research on children’s lexical processing and inferences in word learning.

## Conflict of Interest Statement

The authors declare that the research was conducted in the absence of any commercial or financial relationships that could be construed as a potential conflict of interest.

## Footnotes

1 The study was approved by the Child Subjects Committee of the department.

2 Familiar objects which were used in the warm-up trials were: horse, tiger, elephant, giraffe, zebra, cow, frog, and monkey.

3 Note that not all children contributed data to both sessions: 13 children contributed only to the LF session and five children contributed only to the HF session. Therefore, the analyses comparing the children’s object selection against chance level were run on different numbers of children than the (within-subjects) analysis comparing the children’s object selection across conditions.
