# Supplementary material for: Children’s level of word knowledge predicts their exclusion of familiar objects as referents of novel words
Source: Front Psychol. 2015 Aug 11;6:1200. doi: 10.3389/fpsyg.2015.01200 (PMC4531215; doi:10.3389/fpsyg.2015.01200)
Supplement: Supplementary file 1 [file Supplementary_Material.PDF]

SUPPLEMENTARY MATERIAL:

Pretest to determine the material used in the main study

The rationale of the pre-test was to determine the familiar objects for the main study. Specifically, 6 familiar objects for which children from 2 to 4 years of age know the labels very well, and 18 familiar objects for which 2- to 4-year-olds know the labels less well (6 per age group) were needed.

Well known object labels

In order to determine highly familiar object labels, we followed the procedure used by Mervis and Bertrand (1994) and other researchers and first compiled a list of highly familiar object labels for concrete inanimate objects by taking nouns 1) from two German vocabulary tests for 24-month-olds (SETK-2, Grimm, 2000 and ELFRA, Grimm & Doil, 2000) and 2) from the transcriptions of five German 20-month-olds from the CHILDES Database (MacWhinney, 2000): Leo (Behrens, 2006); Kersten and Simone (Miller 1979); and Pauline from the Rigol Corpus. This list comprised 97 labels.

In the next step, a shortlist was created, which comprised those object labels that according to the norming samples of the SETK and/or the ELFRA are in the productive vocabularies of more than 80% of 24-month-old children (16 items, see Table A). From this shortlist we chose toy exemplars of the following kinds: car, shoe, chair, banana, apple, and tree (cf., Law II and Edwards 2014; Mervis and Bertrand 1994 for a similar method).

Note that these six objects were used for all age groups in the main study. The rationale for this was that if these are highly familiar labels for 2-year-olds, they are also highly familiar to 3- and 4-year-olds.

Less known labels

The less well known object labels were determined separately for each age group. Overall, six familiar objects were identified for which children know the labels but do so less well based on the assumption that words in children's receptive vocabularies are less well known than words in children’s productive vocabularies (cf., McMurray, Horst, and Samuelson 2012). Different methods were used to determine the less well known object labels for 2-year-olds and 3- and 4-year-olds.

2-year-olds. For the 2-year-olds a parental questionnaire was developed. The questionnaire (see Table B) comprised 42 labels for concrete inanimate objects. Of these 42 items, 15 were taken from the shortlist of well-known labels. These items served as fillers in order to avoid biased answering or annoyance on part of the parents. The remaining 27 items were chosen from the longlist of words described above. These 27 items were chosen based on the German norming samples and/or the data of our corpus-analyses indicated that 20- to 24-month-old children would rarely produce them.

Forty parents of 24-month-old children were asked to judge whether and how often their children produce or comprehend each of the 42 labels in the questionnaire. In addition, the parents were asked to estimate how frequently they themselves use each of the labels. Based on parents’ answers we chose toy exemplars of the following kinds with less well known labels for the 2-year-olds: mushroom, saw, hammer, sponge, brush, and scoop.

3-year-olds. In order to identify object labels that 3-year-olds understand well but which they are unlikely to produce, a pretest with 3-year-olds was conducted. Specifically, the production and comprehension of 14 labels for concrete objects (see Table C) was

assessed for ten 3-year-olds (mean age 3;2,6). First, the children were familiarized with all objects. That is, the child and the experimenter (E) played with each object for about half a minute one after the other. E did not label any object during this familiarization phase but said things like "that's fun" or "now it's your turn". Subsequently, the children's productive and receptive knowledge of the objects' labels was tested using a picture book with photographs of the objects. Each page in the picture book showed photographs of three of the target objects, one additional familiar object and one novel object. For each page, the experimenter first encouraged the child to name the objects in a very general way (e.g., "What do you see on this page?"). Any object label that the children provided was coded as "spontaneous labelling". (If children had labelled an object during the prior familiarization phase, this was also coded as "spontaneous labelling".) Subsequently, E pointed at each of the familiar objects on a page that the child had not yet labelled and asked the child, "Do you know what that is?" Any object label provided in response to this specific label request was coded as "labelled upon request". Finally, after E and the child had finished the picture book, the book was read a second time in order to test children's comprehension of all remaining labels that the child had not produced in the first round. That is, for each page, E labelled the objects that the child had not named herself and asked the child to point to the corresponding picture. Based on the children's naming and comprehension, we chose toy exemplars of the following kinds with less well known labels for the 3-year-olds: tongs, nail file, spanner, hanger, top, and lock.

4-year-olds. The same procedure was used to determine less well known labels for 4-year-olds. Twelve 4-year-olds (mean age 4;6,14) participated in the pretest. A set of 17 objects (see Table D) and a picture book with corresponding photographs were used. Based on the children's naming and comprehension, we chose toy exemplars of the following kinds with less well known labels for the 4-year-olds: strainer, thermometer, microphone, can opener, corkscrew, and tweezers.

References

Behrens, Heike. 2006. "The Input-Output Relationship in First Language Acquisition." *Language and Cognitive Processes*, 21: 2-24.

Grimm, Hannelore. 2000. "*Sprachentwicklungstest für zweijährige Kinder (SETK-2): Diagnose rezeptiver und produktiver Sprachverarbeitungsfähigkeiten.*" Göttingen. Hogrefe.

Grimm, Hannelore, & Doil, Hildegard. 2000. "*ELFRA - Elternfragebögen für die Früherkennung von Risikokindern*." Göttingen, Hogrefe.

Law II, Franzo, and Jan R. Edwards. 2014. "Effects of Vocabulary Size on Online Lexical Processing by Preschoolers." *Language Learning and Development*, 1–25. doi:10.1080/15475441.2014.961066.

MacWhinney, Brian. 2000. "The CHILDES Database: Tools for Analyzing Talk, Vol 2: The database."

McMurray, Bob, Jessica S. Horst, and Larissa K. Samuelson. 2012. "Word Learning Emerges from the Interaction of Online Referent Selection and Slow Associative Learning." *Psychological Review* 119 (4): 831–77. doi:10.1037/a0029872.

Mervis, Carolyn B., and Jacquelyn Bertrand. 1994. "Acquisition of the Novel Name–nameless Category (N3C) Principle." *Child Development* 65 (6): 1646–62.

Miller, Dr Max. 1979. "A Quantitative Analysis of the Early Linguistic Development of Meike and Simone." In *The Logic of Language Development in Early Childhood*, 25–34. Springer Series in Language and Communication 3. Springer Berlin Heidelberg. http://link.springer.com/chapter/10.1007/978-3-642-67408-2_3.

Table A

Short list of labels for concrete inanimate objects in the productive vocabulary of more than 80% of 24-month-old children.

Label (German)	Translation (English)	Item difficulty SETK	Item difficulty ELFRA
**Apfel**	apple	86.4	85.61
**Auto**	car	97.9	97.05
Ball	ball	97.6	81.55
**Banane**	banana		83.6
**Baum**	tree	85.98	84.3
Bett	bed		90.7
Buch*	book	55.35	85.7
Eis	ice cream		89.3
Haus	house	89.67	83.6
Hose	trousers		82.9
Keks	cookie		84.3
Löffel	spoon		80.00
**Schuh**	shoe		87.9
**Stuhl**	chair	90.41	79.3
Tisch	table		81.4
Uhr	watch	88.56	86.4

Note: The items labelled in bold were chosen because in both norming studies more than 80% of the children had these labels in their productive vocabulary. "Haus" (house) was not chosen because there was no miniature exemplar available. "Uhr" (clock/watch/alarm) was not chosen because it was unclear whether children are more likely to know this label for watches or for alarm clocks or both. Of the remaining labels "Banane" (banana) and "Schuh" (shoe) were chosen for practical reasons.

* this item was not used as a filler in the parental questionnaire that was used in order to determine labels that are likely to be in 24-month-olds receptive vocabularies

Table B
Short list of labels for concrete inanimate objects likely to be in the receptive vocabulary of 24-month-old children.

Label (German)	Translation (English)	Item difficulty SETK	Item difficulty ELFRA	In corpus
Boot	boat			Leo (6), Simone (1)
Brille	glasses		75.7	Leo (19)
Brot	bread			Leo (20), Simone (1)
**Bürste**	hairbrush	33.21	65.7	Leo (1)
Fahrrad	bike			Leo (1), Simone (7)
**Hammer**	hammer			Simone (3)
Handschuh	gloves		60.0	Leo (3)
Kamm	comb	35.06	51.4	
Kanne*	can			
**Kelle**	ladle			Leo (10)
Kerze	candle			Leo (48), Simone (5)
Knopf	button			Simone (7)
Pfanne	pan			Simone (2)
**Pilz***	mushroom			
Pinsel*	brush			
Ring	ring			Pauline (1), Simone (1)
**Säge**	saw			Leo (1)
Schaufel	shovel			Leo (5)
Schrank	wardrobe		55.0	Leo (7)
**Schwamm**	sponge			Simone (3)
Sieb*	strainer			
Sofa	sofa		62.1	Leo (2),
Stift	crayon	79.34	59.3	Leo (4), Kerstin (5)
Tasche	bag			Kerstin (2), Pauline (2), Simone (5)
Teller	plate			Simone (2)
Topf	pot			Leo (2), Simone (5)
Zange*	tongs			

Note. The bold faced items were chosen based on the parents' judgment.

* These tools and kitchen utensils were added to the questionnaire although the labels were not attested in the references. Nevertheless the corresponding objects are less familiar to children.

Table C
List of labels which were pre-tested to serve as less well known objects in the main study for 3-year-olds

Label (German)	Translation (English)	Spontaneously labeled	Labeled after request	Comprehen-sion	Unknown
**Bügel**	coat hanger	1	3	3	3
Feile	file	2		4	4
Fernglas	looking glass	1	1	2	6
Globus	globe	1		3	6
Korkenzieher	corkscrew			4	6
**Kreisel**	spin top			9	1
Nuss	nut	3		3	4
Pinzette	tweezers				10
**Schloss**	lock		1	6	3
**Schrauben-schlüssel***	wrench			5	4
**Schwamm**	sponge	1		6	3
Sieb	strainer			4	6
**Zange**	tongs	2	1	5	2
Zollstock	folding ruler			4	5

Note: The items labelled in bold were chosen because they appeared to be the most likely candidates for words in 3-year-olds receptive vocabularies.

* this item was tested on 9 children only

Table D

List of labels which were pre-tested to serve as less well known objects in the main study for 4-year-olds

Label (German)	Translation (English)	Spontaneously labeled	Labeled after request	Comprehen-sion	Unknown
Batterie	battery	8	1	3	
**Dosenöffner**	can opener	1		9	2
Federball	badminton	4	1	6	1
Feile	file			3	9
Globus	globe	3		4	5
**Korkenzieher**	cork screw	1		10	1
Lippenstift	lipstick	7		5	
Lupe	magnifier	7		4	1
**Mikrophon**	microphone	1		11	
Mikroskop	microscope			4	8
Mundharmo-nika	mouth organ	3		6	3
**Pinzette**	tweezers			9	3
Rollschuh	inline skates	10		1	1
**Sieb**	strainer	2	1	8	1
**Thermometer**	thermometer	1	1	10	
Trichter	funnel		1	7	4
Zollstock	folding ruler			8	4

Note: The items labelled in bold were chosen because they appeared to be the most likely candidates for words in 4-year-olds receptive vocabularies.

Table E
List of excluded trials per type, session, age group, number of children and object selection.

Trial type	Number of trials	Number of trials per session	Number of trials per age group	Number of children (trials per child)	Object selection
No familiar object word knowledge	76	2 in HF	2 in 2yo	1	1 novel<br>1 familiar
		74 in LF	39 in 2yo	17 (1 to 4)	24 novel<br>15 familiar
			14 in 3yo	9 (1 to 2)	6 novel<br>8 familiar
			21 in 4yo	10 (1 to 4)	10 novel<br>11 familiar
Novel-labelled-trials	18	10 in HF	2 in 2yo	1	1 novel<br>1 familiar
			1 in 3yo	1	1 novel<br>0 familiar
			7 in 4yo	5 (1 to 2)	6 novel<br>1 familiar
		8 in LF	3 in 3yo	1	2 novel<br>1 familiar
			5 in 4yo	5	2 novel<br>3 familiar

Note: Labels for novel objects included *at the bike / bike / lamp for a bike* (N2), *candle / magnifier* (N3), *number / horseshoe* (N7), *christmas tree* (N8), *spin top* (N10), *toy for a baby* (N11).
Pictures of the novel objects used in the study can be seen below.

Table F
Novel objects used in the study.

**N1**	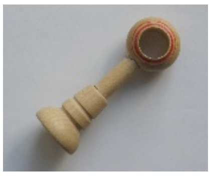	**N2**	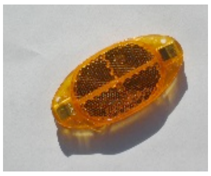	**N3**	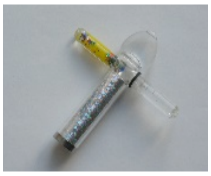	**N4**	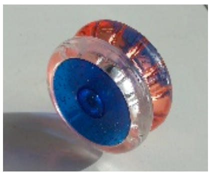
**N5**	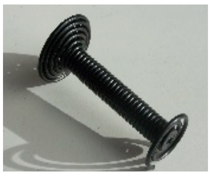	**N6**	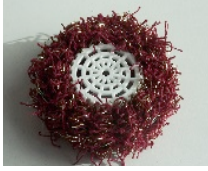	**N7**	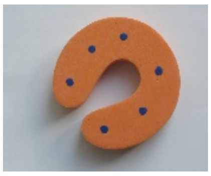	**N8**	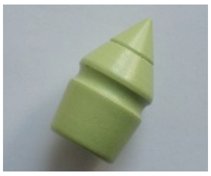
**N9**	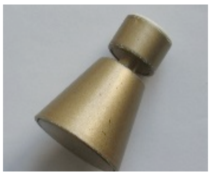	**N10**	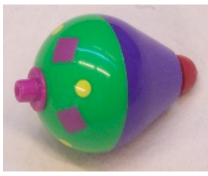	**N11**	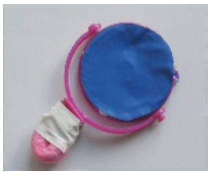	**N12**	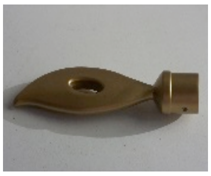
